# Root-Securing and Brain-Fortifying Liquid Upregulates Caveolin-1 in Cell Model with Alzheimer's Disease through Inhibiting Tau Phosphorylation

**DOI:** 10.1155/2017/6248351

**Published:** 2017-10-16

**Authors:** Depei Yuan, Chuhua Zeng, Qianfeng Chen, Fengjie Wang, Lin Yuan, Yaoqian Zhu, Ziyang Shu, Ning Chen

**Affiliations:** ^1^College of Medicine, Hubei University for Nationalities, Enshi 445000, China; ^2^Tianjiu Research and Development Center for Exercise Nutrition and Foods, College of Health Science, Wuhan Sports University, Wuhan 430079, China

## Abstract

In order to explore the effect of root-securing and brain-fortifying Liquid- (RSBFL-) mediated caveolin-1 (CAV-1) on phosphorylation of Tau protein and to uncover underlying mechanisms of RSBFL for the prevention and treatment of Alzheimer's disease (AD), hippocampal neurons isolated from neonatal SD rats and cultured in DMEM-F12 medium were induced by exogenous A*β*1–42 to establish a cell model with AD. Meanwhile, pEGFP-C1-CAV1 and CAV1-shRNA plasmids were transfected into hippocampal neurons for CAV-1 overexpression and silence, respectively. The serum containing RSBFL was prepared for the intervention of AD model cells. The expression of CAV-1, GSK-3*β*, and p-Tau in normal hippocampal neurons and AD model cells in the presence of serum containing RSBFL was evaluated. The model hippocampal neurons with AD induced by A*β*1–42 revealed an obvious CAV-1 inhibition, enhanced GSK-3*β* activity, and abnormal Tau phosphorylation. In contrast, the treatment with serum containing RSBFL could upregulate CAV-1 in AD hippocampal neurons (*P* < 0.05) with improved p-GSK-3β^Ser9^ and reduced p-GSK-3*β*^Tyr216^ (*P* < 0.01), as well as suppressed abnormal phosphorylation of Tau protein. Therefore, RSBFL has an excellent protective effect on hippocampal neurons through increasing CAV-1 expression, inhibiting GSK-3*β* activity, and reducing excessive abnormal phosphorylation of Tau protein.

## 1. Introduction

Alzheimer's disease (AD) is a progressive degenerative disease of central nervous system and has two major characteristics including the formation of neurofibrillary tangles (NFTs) from the aggregates of excessively phosphorylated Tau protein in neurons [1] and the formation of senile plaques (SPs) from the aggregates of *β*-amyloid (A*β*) outside neurons. The number of NFTs is closely correlated with dementia degree of AD patients [2]. In addition, excessive abnormal phosphorylation of Tau is mainly regulated by glycogen synthase kinase-3*β* (GSK-3*β*), Cdk5, MAPK, and other protein kinases. Thr231 and Ser396 are recognized as the major phosphorylation sites of Tau in AD. Moreover, GSK-3*β* reveals the highest affinity and the strongest phosphorylation for Tau protein. Currently, a number of studies confirm that the activation of GSK-3*β* can obviously promote the phosphorylation of Tau [3, 4]. Chinese medicine has gained extensive attention for the prevention and treatment of AD, which will be beneficial for development and utilization of natural products for AD. However, the underlying mechanisms of Chinese herbs for the prevention and treatment of AD are still unclear. Therefore, GSK-3*β* could be considered as the target to explore the possible mechanisms of AD during the application of traditional Chinese medicine.

Root-securing and brain-fortifying liquid (RSBFL) composed of* Codonopsis tangshen*,* Lycium barbarum*,* Poria cocos*,* Ziziphus jujuba *Mill. var.* spinosa*, and* Crataegus pinnatifida *Bunge has the multiple functions of scavenging free radicals [5], modulating immune [6], and protecting brain nerves [7] for contributing to the prevention and treatment of AD due to the action of polysaccharides from* Codonopsis tangshen* and* Lycium barbarum*. As a famous formula with application history of more than 20 years for fortifying brain and boosting intelligence of RSBFL, many corresponding investigations have been conducted to validate its functions and treatment efficacy. The polysaccharides from* Codonopsis tangshen* in this formula can execute antioxidant and antiaging effects in D-gal-induced aging mice [8] and can rescue the impaired memory capacity of the mice due to the toxicity from lead and scopolamine through mitigating lipid peroxidation and accelerating the clearance of free radicals [9]. Similarly, the polysaccharides from* Lycium barbarum* can reduce methylmercury-induced hippocampal neural stem cell injury and promote the differentiation of hippocampal neural stem cells into neurons and the growth of neurons [10]. Our previous studies have confirmed that RSBFL can obviously improve the morphology and density of dendritic spines, promote the expression of CAV-1, and suppress excessive abnormal phosphorylation of Tau protein in hippocampal neurons [11]. Similarly, previous reports have also demonstrated that the impairment of learning and memory capacity in AD patients has a direct correlation with loss and apoptosis of neurons in cerebral cortex and hippocampal regions [12]. However, the underlying molecular mechanisms still need to be further explored. In the present study, pEGFP-C1-CAV1 transfection and shRNA-CAV1 gene silencing in hippocampal neuron cell model with AD subjected to exogenous A*β*1–42 induction were applied to uncover the molecular mechanisms of RSBFL for the prevention and treatment of AD and the improvement of learning and memory capacity through attenuating oxidative stress and alleviating free radical damage.

## 2. Materials and Methods

### 2.1. Isolation, Identification, and Cultivation of Hippocampal Neurons

Neonatal specific pathogen-free (SPF) Sprague Dawley (SD) rats of the age of 24 h were provided by the Experimental Animal Center of Three Gorges University in China. According to the methods from previous studies [13], SD rats were sacrificed under sterilization condition, the whole brain was carefully harvested, and hippocampal tissue was segregated and placed in ice-chilled D-Hanks solution. After removing microvessels and cutting into small pieces, the small pieces of hippocampal tissues were added with 0.25% trypsin for digestion at 37°C for 20 min until adding fetal bovine serum (FBS) to terminate digestion. After filtration, cell suspension was collected and cultivated in a well-prepared 6-well plate at the cell population of 7 × 10^5^ cells/well in DMEM-F12 medium with 10% FBS. Then, neuronal cells after being adhered to the bottom of plate were cultivated in fresh DMEM-F12 medium with 10% FBS, 2% B27, 2 mM L-glutamine, 100 U/ml penicillin, 100 U/ml streptomycin, and neurobasal-A at 37°C and 5% CO_2_ in a humidified incubation chamber. The cultivation medium was replaced by fresh medium every 3 days. The cultured hippocampal neurons were identified by immunofluorescence and Western blot.

### 2.2. Construction of pEGFP-C1-CAV1 Plasmid

According to gene sequence of CAV-1 from rats in Gene Bank (156–692,536 bp), the primers of CAV-1 for polymerase chain reaction (PCR) amplification were synthesized in Shanghai Sangon Biotech Co., Ltd. (Shanghai, China). Total RNA was extracted from rat brain through Total RNA Extraction Kit (Magen, Guangzhou, China) according to the manufacturer's instructions. The reverse transcription reaction of the extracted RNA was conducted by using reverse transcription kit (GeneCopoeia, Inc., Rockville, MD, USA) to obtain cDNA according to the manufacturer's protocols. Totally 30 *μ*L of DNA was purified by using Universal DNA Purification Extraction Kit (TIANGEN Biotech Inc., Beijing, China) according to the manufacturer's instructions. The pEGFP-C1 vector and purified CAV-1 gene product were subjected to double digestion by restriction endonucleases EcoR I and Sal I. The digested products were purified and harvested by electrophoresis. The CAV-1 gene was spliced into linear pEGFP-C1 vector to obtain pEGFP-C1-CAV1. The recombinant pEGFP-C1-CAV1 plasmid was subjected to extraction, double digestion by EcoR I and Sal I, identification, and sequence analysis. The validated pEGFP-C1-CAV1 plasmid was transferred into hippocampal neuronal cells for coming experiments.

### 2.3. Construction of CAV1-shRNA Vector

According to the previous report [14], the hairpin structure of rat CAV-1 shRNA includes a sense strand of GACGTGGTCAAGATTGACT and an antisense strand of AGTCAATCTTGACCACGTC, as well as a loop of TCTCTTGAA. The plasmid was constructed through lentiviral transfection. The CAV1-shRNA, shuttle plasmid LV3-NC, and lentiviral packaging plasmid at appropriate proportion were added for virus packaging. The solution with packaged virus was collected for future use. The titer of the virus was determined by counting produced foci as a result of viral infection under a fluorescent microscope. Subjected to repeated experiments, the optimal transfection methods and parameters were validated.

### 2.4. Preparation of Serum Containing Drugs and Establishment of Cell Model with AD

#### 2.4.1. The Preparation of Serum Containing Drugs

Totally 60 SPF SD rats of the age of 4 months were randomly divided into three groups: normal control, RSBFL, and donepezil groups with 20 rats in each group. RSBFL and donepezil were purchased from the Affiliated Hospital of Hubei University for Nationalities and Eisai China Inc. The rats were subjected to regular gavage with RSBFL and donepezil at the doses of 31 g/kg·bw (equivalent to the herb amount in the formula) and 2.25 g/kg·bw every day, respectively. The rats from normal control group were subjected to identical volume of distilled water every day. After drug administration for 1 week, the sera were collected from abdominal aortic artery of the rats for future use.

#### 2.4.2. The Establishment of Cell Model with AD

A*β*1–42 (Sigma, St. Louis, MO, USA) was diluted with distilled water and incubated in 37°C water bath for 3 d to prepare the aggregates of A*β*1–42 to 125 mmol/L as the stock solution. Then the stock solution was added to the cultured hippocampal neurons to a final concentration of 2.5 mmol/L. The viability of hippocampal neurons subjected to A*β*1–42 treatment was evaluated by MTT assay and A*β*1–42-induced p-Tau level in hippocampal neurons was also determined.

### 2.5. The Drug Intervention of Model Cells with AD

The established model cells with AD were cultivated in DMEM-F12 medium with 10% FBS, 2% B27, 2 mM L-glutamine, 100 U/ml penicillin, 100 U/ml streptomycin, and neurobasal-A at 37°C and 5% CO_2_ in a humidified incubation chamber and then divided into 5 groups including normal neuron cultivation medium as the normal control (NC), normal neuron cultivation medium plus 10% serum without any drugs as the blank (B), normal neuron cultivation medium coupled with A*β*_1–42_ plus 10% RSBFL-containing serum (RSBFL), normal neuron cultivation medium coupled with A*β*_1–42_ plus 10% donepezil-containing serum (Don), and normal neuron cultivation medium coupled with A*β*_1–42_ plus 10% serum without any drugs as the AD model (Mod) groups. After intervention by RSBFL- or donepezil-containing serum for 12 h, the cells were harvested for the evaluation of CAV-1, GSK-3*β*, and Tau protein expression by Western blot.

### 2.6. Western Blot

The extracted protein from hippocampal neurons in each group was analyzed by Western blot. The protein was quantified by BCA Protein Assay Kit (Boster Biological Engineering Co., Ltd., Wuhan, China) according to BCA (bicinchoninic acid) method. The proteins were separated in 4–6% sodium dodecyl sulfate polyacrylamide gel electrophoresis (SDS-PAGE) and then transferred on a PVDF membrane. The membrane was blocked with 5% nonfat milk. The target protein was probed with p-Tau (Thr231) and caveolin-1 (Abcam, Cambridge, UK) and p-Tau (Ser396), p-GSK-3*β* (Ser9), p-GSK-3*β* (Tyr216), and GAPDH (Santa Cruz Biotechnology, Santa Cruz, CA, USA), as well as sequential secondary antibody. ECL luminescence was used for imaging, and finally the optical density of the band was analyzed by gel imaging system (Bio-Rad Laboratories, Hercules, CA, USA).

### 2.7. Statistical Analysis

Image analysis system Image-Pro plus software was used for the semiquantitative analysis of the results from Western blot and immunofluorescence. All resultant data were expressed as mean ± standard deviation (M ± SD). The data comparison between groups was conducted by using single factor analysis of variance (one-way ANOVA) through SPSS 18.0 software. The statistically significant difference and very statistically significant difference were considered at* P* < 0.05 and* P* < 0.01, respectively.

## 3. Results

### 3.1. Construction and Optimal Transfection of pEGFP-C1-CAV1 Plasmid

**Figure 1:**
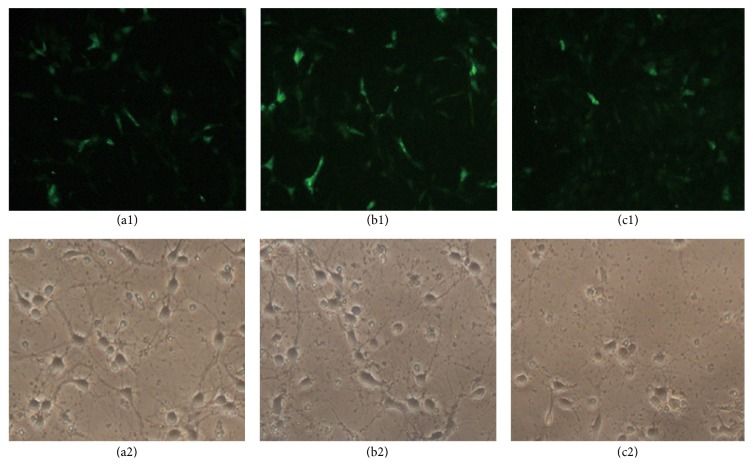
The optimal transfection screening for pEGFP-C1-CAV1 plasmid in hippocampal neurons under the plasmid-liposome at the ratio of 2.5 : 3.75 (a1), 5 : 7.5 (b1), and 7.5 : 11.25 (c1) was evaluated by EGFP fluorescent intensity, respectively. Meanwhile, the optimal transfection screening for pEGFP-C1-CAV1 plasmid in hippocampal neurons under the plasmid-liposome at the ratio of 2.5 : 3.75 (a2), 5 : 7.5 (b2), and 7.5 : 11.25 (c2) was also evaluated by the morphology and nerve network of hippocampal neurons, respectively.

The recombinant pEGFP-C1-CAV1 plasmid was successfully constructed according to the above stated methods. After transfecting recombinant pEGFP-C1-CAV1 plasmid in hippocampal neurons, the transfection at the ratio of 5 *μ*L plasmid to 7.5 *μ*L liposome could result in the strongest fluorescence intensity in transfected cells at the largest population (Figure 1(b1)), which was followed by the ratios of 2.5 *μ*L plasmid to 3.75 *μ*L liposome (Figure 1(a1)) and 7.5 *μ*L plasmid to 11.25 *μ*L liposome, as shown in the weakest fluorescence intensity and large amount of cell death (Figure 1(c1)). The transfection at the ratio of 5 *μ*L plasmid to 7.5 *μ*L liposome could result in the best cell status, obvious halo and clear protrusion network (Figure 1(b2)). Although the transfection at the ratio of 2.5 *μ*L plasmid to 3.75 *μ*L liposome could also result in the better cell status and diopter, the nerve network was weaker (Figure 1(a2)). The transfection at the ratio of 7.5 *μ*L plasmid to 11.25 *μ*L liposome could result in the weakest cell status, interrupted nerve network and disappeared protrusions and halo, and even largely reduced neuronal number (Figure 1(c2)). Therefore, the optimal transfection condition should be of the ratio of 5 *μ*L plasmid to 7.5 *μ*L liposome.

### 3.2. Construction and Expression of CAV1-shRNA Plasmid

**Figure 2:**
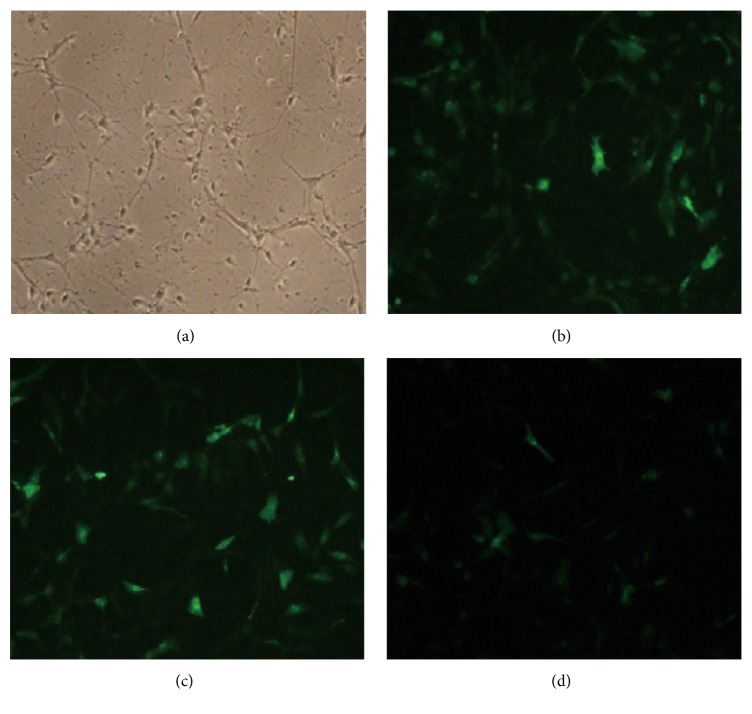
The transfection efficiency of CAV1-shRNA under the conditions with various MOI of 100 (b), 10 (c), and 1 (d) evaluated by EGFP fluorescent intensity and the optimal transfection efficiency of CAV1-shRNA evaluated by morphology, number, and nerve structure of hippocampal neurons (a), respectively.

As shown in Figure 2, after 12 h transfection, all hippocampal neurons revealed excellent status, good diopter, clear protrusions, and dense network (Figure 2(a)), and there was no significant difference when compared with the cells without transfection, suggesting that lentivirus had no obvious toxicity to hippocampal neurons at the current status. These results confirmed that CAV1-shRNA was successfully constructed by lentiviral transfection and had sufficient infection capacity (titer 9 × 10^8^ TU/mL) with the best MOI value of 10. Meanwhile, the constructed plasmid revealed excellent expression in targeted hippocampal neurons. The MOI of 100 could lead to the highest GFP expression due to the strongest fluorescence intensity and the largest amount of fluorescent cells (Figure 2(b)), suggesting the highest transfection efficiency. Similarly, the MOI of 10 revealed no significant difference in transfection efficiency (Figure 2(c)) when compared with the MOI of 100. In contrast, the MOI of 1 exhibited an obviously insufficient biological activity of viral particles due to the weakest EGFP expression and fluorescence intensity, and the smallest fluorescent cell population (Figure 2(d)), indicating the low transfection efficiency.

### 3.3. AD Cell Model Establishment through A*β*1–42 Stimulation

**Figure 3:**
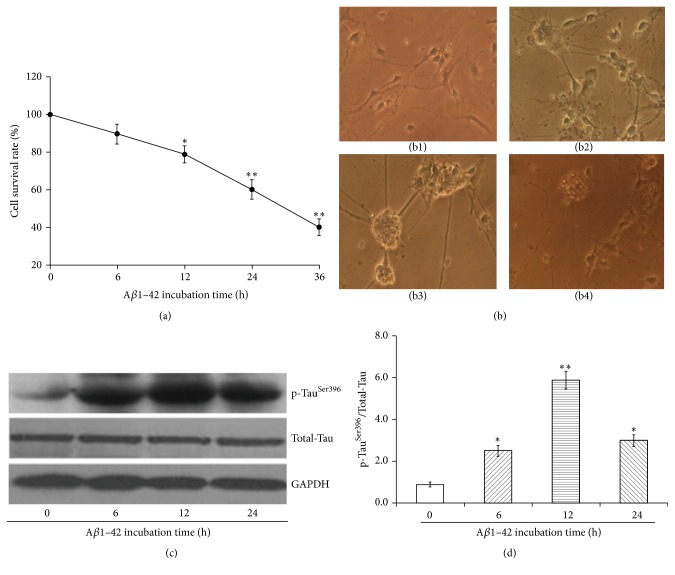
The cell growth inhibition (a), characteristic morphological changes (b), and expression of p-Tau^Ser396^ protein (c, d) of hippocampal neurons in the presence of A*β*1–42 induction (^*∗*^*P* < 0.05, ^*∗∗*^*P* < 0.01).

As shown in Figure 3(a), after A*β*1–42 at a final concentration of 2.5 mmol/L was incubated with primary hippocampal neurons, the viability of hippocampal neurons revealed a gradual decrease as the extension of stimulation time in a time-dependent manner. The viability of hippocampal neurons was 79% in the presence of 2.5 mmol/L A*β*1–42 at the incubation time of 12 h, while the viability of hippocampal neurons was reduced to less than 60% during the incubation time of 24–36 h (Figure 3(a)).

A*β*1–42 could induce the damage of primary hippocampal neurons as the increase of induction time. The hippocampal neurons at normal conditions revealed good refraction, strong three-dimensional sense, full body volume, obvious halo around cell body, continuous extending protrusions with more branches, and interconnected and intertwined network (Figure 3(b1)). After A*β*1–42 induction for 6 h, the boundary of cells became blur and began to reveal aggregation and retraction; meanwhile, the protrusions of hippocampal neurons were reduced (Figure 3(b2)). After A*β*1–42 induction for 12 h, obvious cell shrinkage, aggregation, interrupted cell connection, and partial necrotic cells were observed (Figure 3(b3)). After A*β*1–42 induction for 24 h, serious damage of hippocampal neurons, reduced or even disappeared halo, more cell death, and further reduced cell junctions of hippocampal neurons were observed (Figure 3(b4)).

As the extension of A*β*1–42 incubation time, the expression of p-Tau^Ser396^ protein in hippocampal neurons increased and reached up to the highest level at the induction time of 12 h; on the contrary, the expression of p-Tau^Ser396^ protein in hippocampal neurons revealed an obvious reduction at the induction time of 24, which may be due to the necrosis of hippocampal neurons (Figures 3(c) and 3(d)).

Based on the above comprehensive consideration, the final concentration of A*β*1–42 should be 2.5 mmol/L. The induction temperature of 37°C and induction duration of 72 h could lead to the aggregation of hippocampal neurons and successful establishment of AD cell model at the induction time of 12 h.

### 3.4. Effect of RSBFL on Expression of CAV-1, p-Tau, and GSK-3*β* in Hippocampal Neurons

**Figure 4:**
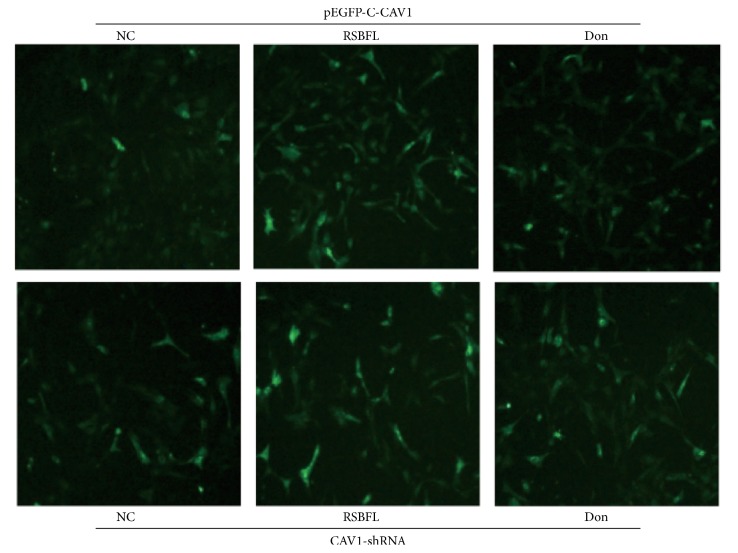
The pEGFP-C1-CAV1 expression in hippocampal neurons subjected to the treatments with sera containing RSBFL and donepezil in the presence and absence of CAV1-shRNA.

Hippocampal neuronal cells subjected to blank control, RSBFL, and donepezil treatments at the optimal dose of 10% in prepared sera were cultured in the presence of CAV1-shRNA and pEGFP-C1-CAV1, respectively. In addition, hippocampal neuronal cells without any treatments were used as the normal control. As shown in Figure 4, after hippocampal neuron cultivation for 72 h, the expression of GFP was present in hippocampal neurons transfected with recombinant pEGFP-C1-CAV1 plasmid. Although each group revealed high fluorescence intensity and large amount of fluorescent cells as well as high transfection efficiency, the hippocampal neuronal cells from RSBFL groups revealed excellent fluorescent intensity and more fluorescent cells.

**Figure 5:**
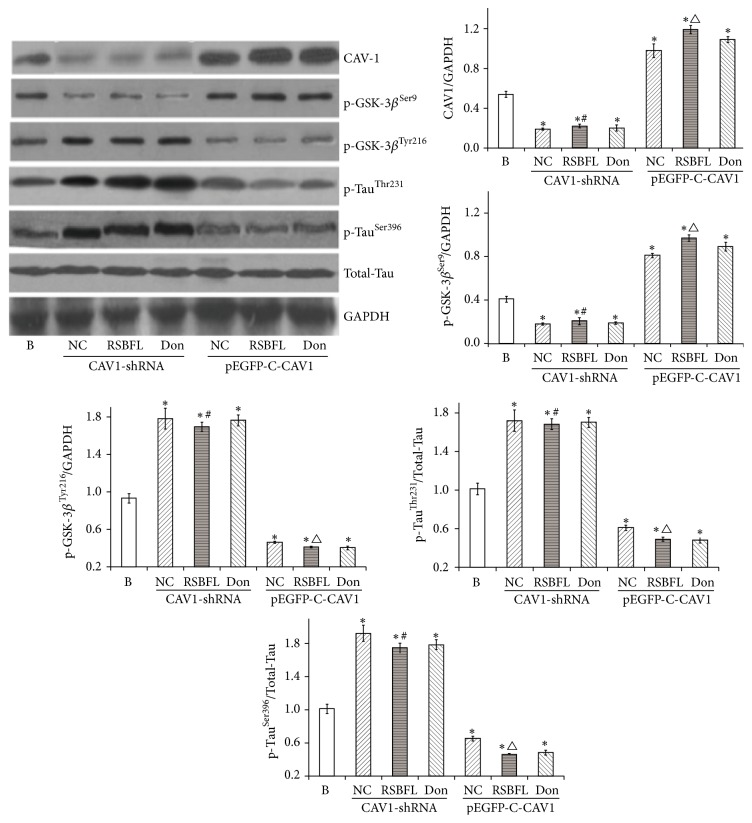
The expression of CAV1, p-Tau, and GSK-3*β* in hippocampal neurons subjected to the treatments with sera containing RSBFL and donepezil in the presence and absence of CAV1-shRNA. ^*∗*^*P* < 0.05 relative to B group; ^#^*P* < 0.05 relative to NC group in CAV1-shRNA condition; ^△^*P* < 0.05 relative to NC group in pEGFP-CAV1 condition.

Similarly, as shown in Figure 5, after hippocampal neuron cultivation for 72 h, the hippocampal neurons transfected with CAV1-shRNA revealed the silence of CAV-1 gene and the significantly lower expression of CAV-1 and p-GSK-3*β*^Ser9^ (*P* < 0.01), as well as the obviously enhanced expression of p-GSK-3*β*^Tyr216^, p-Tau^Thr231^, and p-Tau^Ser396^. In contrast, hippocampal neurons transfected with pEGFP-C1-CAV1 plasmid exhibited the upregulated CAV-1 and p-GSK-3*β*^Ser9^ (*P* < 0.01) and downregulated p-GSK-3*β*^Tyr216^ (*P* < 0.01), p-Tau^Thr231^ (*P* < 0.05), and p-Tau^Ser396^ (*P* < 0.05) when compared with normal hippocampal neurons.

According to the statistical analysis, CAV-1 gene silence revealed an ideal effect. On the other hand, the serum containing SRBFL could rescue the expression of CAV-1 in the presence of CAV1-shRNA and cause the reduced and enhanced phosphorylation of GSK-3*β* at the sites of Ser9 and Tyr216, respectively, as well as leading to the increased phosphorylation of Tau at the sites of Thr231 and Ser396. These results indicated that RSBFL could promote the expression of CAV-1, inhibit the activity of GSK-3*β*, and reduce the phosphorylation of Tau.

### 3.5. Effect of RSBFL on the Expression of CAV-1, p-Tau, and GSK-3*β* in AD Cell Model

**Figure 6:**
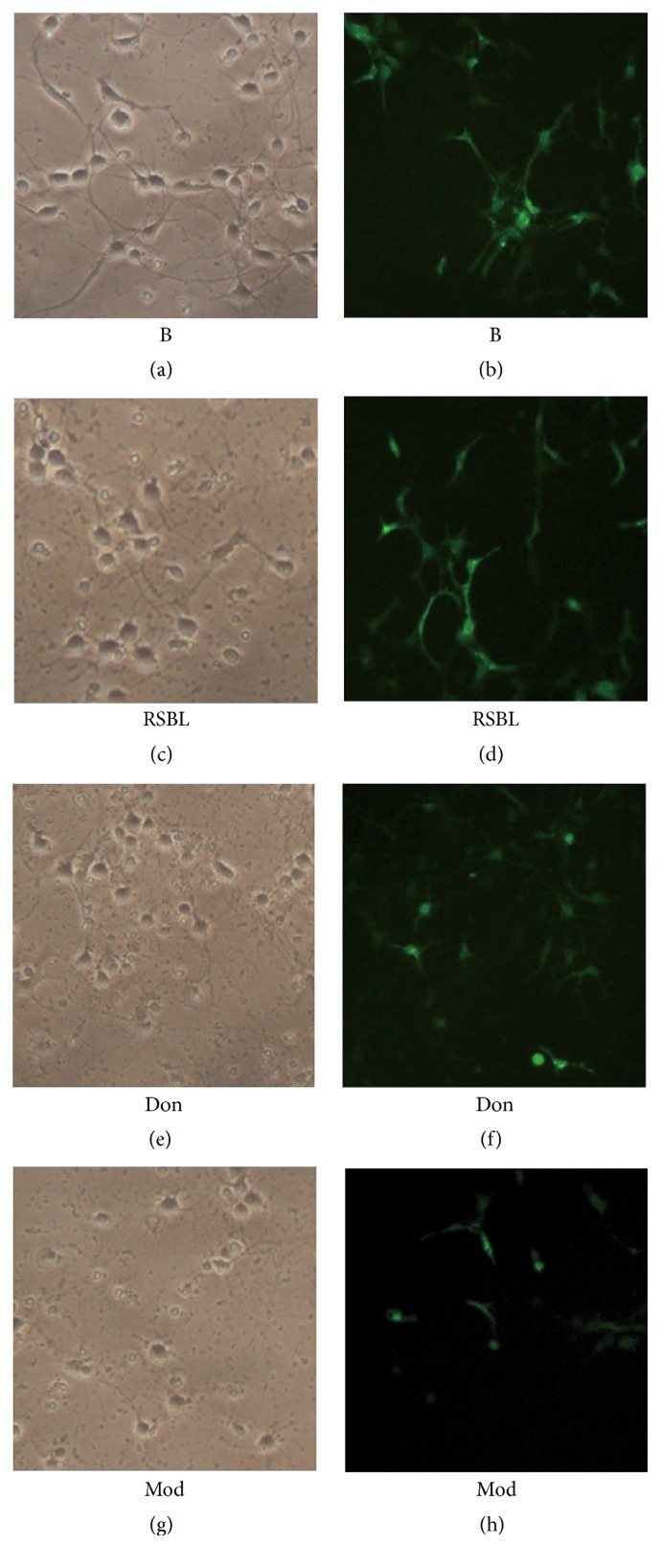
The cell growth status and fluorescence of A*β*1–42-induced hippocampal neurons transfected with pEGFP-C1-CAV1 plasmid after treatments with sera containing RSBFL and donepezil.

After the model establishment for 12 h, cell morphology and GFP fluorescence were observed under an inverted fluorescence microscope. The expression level of CAV-1, GSK-3*β*, and p-Tau in collected hippocampal neurons was evaluated by Western blot. As shown in Figure 6, the morphology of hippocampal neurons at different time points after model establishment for 12 h was observed under an inverted microscope. After 60 h transfection, hippocampal neurons in each group revealed an excellent growth status, longer protrusions, and an intertwined network without significant difference. The strongest GFP fluorescence was observed in blank group (Figure 6(b)). In contrast, hippocampal neurons in RSBFL and donepezil groups revealed a weak growth status and poor fluorescence due to A*β*1–42 induction (Figures 6(c)–6(f)), but the fluorescence in hippocampal neurons from RSBFL group was a little higher than that from donepezil group. In model group, hippocampal neurons exhibited serious disintegration and fluorescent hippocampal neurons were only sporadically visible (Figure 6(h)). Similarly, in the model group, a large number of hippocampal neurons revealed the interrupted protrusions, disintegrated cell bodies, and poor growth status (Figure 6(g)).

**Figure 7:**
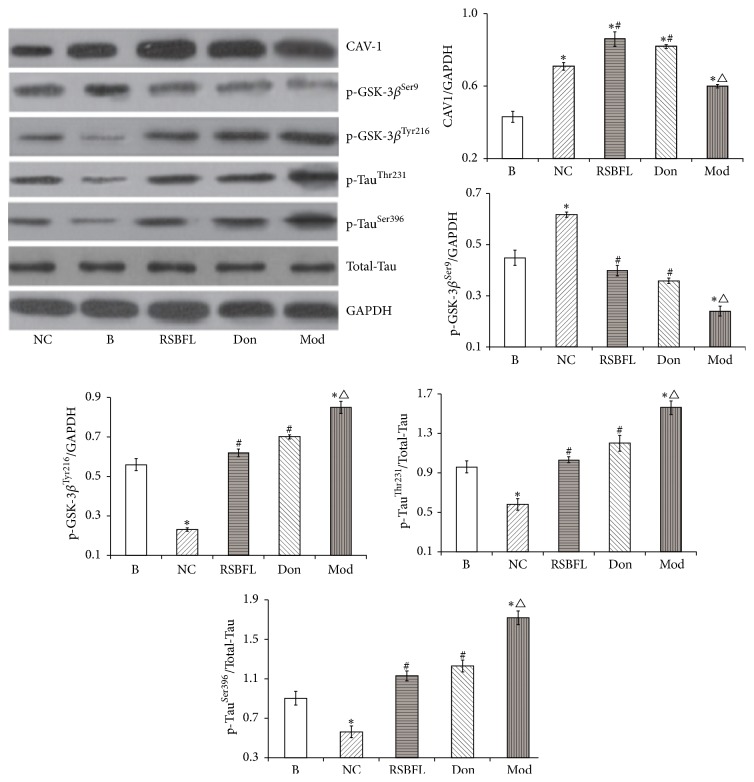
The expression of CAV1, p-Tau, and GSK-3*β* in A*β*1–42-induced hippocampal neuron model with AD after treatments with sera containing RSBFL and donepezil. ^*∗*^*P* < 0.05 relative to B group; ^#^*P* < 0.05 relative to Mod group; ^△^*P* < 0.05 relative to NC group.

Similarly, as shown in Figure 7, hippocampal neurons were transfected with recombinant pEGFP-C1-CAV1 plasmid and induced by A*β*1–42 to establish AD cell model. Compared with the normal group, the expression of CAV-1 and p-GSK-3*β*^Ser9^ revealed a significant increase, but the expression of p-GSK-3*β*^Tyr216^ exhibited a significant reduction (*P* < 0.01). When compared with donepezil and control groups, RSBFL could reinforce the expression of CAV-1 (*P* < 0.05). On the other hand, AD cell model exhibited the decreased expression of CAV-1 (*P* < 0.05) and p-GSK-3*β*^Ser9^ (*P* < 0.01) and the increased expression of p-GSK-3*β*^Tyr216^ (*P* < 0.01), as well as the enhanced phosphorylation of Tau protein at sites of Thr231 and Ser396 (*P* < 0.01).

Based on the above analysis, the intervention using RSBFL and donepezil can improve the phosphorylation of GSK-3*β* at the site of Ser9 and reduce the phosphorylation of GSK-3*β* at the site of Tyr216, as well as suppressing the phosphorylation of Tau protein at Thr231 and Ser9 sites.

## 4. Discussion

Alzheimer's disease is a common neurodegenerative disease, mainly characterized by excessive abnormal phosphorylation of Tau protein, thus leading to the formation of neurofibrillary tangles (NFT) and senile plaques (SP) caused by APP protein metabolic disorders, free radical damage, and nerve cell apoptosis [15], which further results in cognitive impairment. Previous studies have found that oxidative stress plays an increasingly important role in neurodegenerative diseases and aging process and can cause increasing incidence of AD [16] and neurological dysfunction, eventually leading to neuronal apoptosis [17]. Based on previous findings, Tau-associated pathological changes can be observed in the absence of apparent deposition of A*β*. Moreover, the mice with Tau knockout can prevent A*β* toxicity due to the low expression level of Tau [18, 19]. Another study has demonstrated that GSK-3*β* as a critical protein kinase for Tau can phosphorylate APP in cells, thus effectively suppressing the generation of A*β* [20]. These studies suggest that oxidative stress and excessive phosphorylation of Tau protein may play a very important role in the pathogenesis of AD. Currently, Chinese medicine has a certain theoretical and practical foundation in the fight against AD through potential mechanisms of inhibiting neuronal apoptosis, improving learning and memory capacity, attenuating NO-induced neurotoxicity, alleviating oxidative stress, reducing free radical damage, and suppressing inflammation. Therefore, we have tried to uncover the molecular mechanisms of RSBFL for the prevention and treatment of AD through mitigating oxidative stress, reducing free radical damage, and improving learning and memory capacity.

Casal et al. [21] have found that after A*β*25–35 and A*β*1–42 are incubated with rat primary neurons, A*β*25–35 does not reveal the significant change in the morphology of neurons, while A*β*1–42 can result in the morphologic change of neurons. Accordingly, we used sterile triple-distilled water to dilute A*β*1–42 to a final concentration of 2.5 mmol/L. The diluted A*β*1–42 was incubated at 37°C for 72 h to form an aggregation state and was used to induce the damage of hippocampal neurons for 12 h. This cell model can represent the neurotoxic effect during the pathogenesis of AD and the inducing process of abnormal phosphorylation of Tau protein. Thus, this model is a successful AD cell model* in vitro*. In addition, a report has demonstrated a vesicular structure due to the retraction of plasma membrane in cells, and this nest vesicle is also named as Caveolae. CAV is the marker protein of Caveolae [22]. When CAV-1 is overexpressed, *β*-APP and *β*-secretase can be localized to Caveolae and result in decreased *β*-amyloid production, thus suggesting the protective role of CAV-1 [23]. Head et al. [24] have also found that hippocampal regions reveal excessive abnormal phosphorylation of Tau protein and elevated level of A*β* in CAV-1 knockout mice, whose characteristics are similar with the pathological changes of AD; therefore, CAV-1 knockout accelerates neurodegeneration and aging. If mouse primary neurons with CAV-1 knockout are subjected to the transfection of CAV-1 plasmid, the neurons can result in a significant decrease of A*β* level. Trushina et al. [25] have also found that the mice with CAV-1 knockout have abnormal walking, reduced physical activity, and other abnormal phenomena, which is due to Tau hyperphosphorylation in hippocampal tissues. Gaudreault et al. [26] have conducted a comparison between AD patients and normal controls and found downregulated CAV-1 protein in hippocampal regions of AD patients, which is close to the half level in normal persons. In the present study, the silence of CAV-1 gene in normal hippocampal region could result in the downregulation of CAV-1, thus correspondingly leading to the increased phosphorylation levels of Tau at Thr231 and Ser396 sites. In contrast, the overexpression of CAV-1 could reduce the phosphorylation of Tau at Thr231 and Ser396 sites. In our study, RSBFL can promote the upregulation of CAV-1 in hippocampal neurons, which is consistent with our previous animal experiments.

Tau reveals the highest content in microtubule-associated proteins. Under the physiological state, Tau can bind to tubulin to promote the polymerization of tubulin proteins into microtubules and inhibit its dissociation. Tau also can further bind with microtubules to promote their assembly for enhancing their stability [27]. Excessive phosphorylation of Tau protein is due to the interrupted balance between phosphorylation and dephosphorylation. During the phosphorylation process of Tau protein, GSK-3*β* is considered as the most powerful kinase [28]. Previous studies have reported that tangled neurons can be observed in brain tissues of AD patients, and GSK-3*β* presents as an active state. Therefore, the formation of paired helical fiber (PHF) may be correlated with the active state of GSK-3*β*, suggesting that excessive abnormal phosphorylation of Tau protein may be associated with GSK-3*β* activity [29]. Several phosphorylation sites in Tau protein can be activated by GSK-3*β* to result in excessive abnormal phosphorylation [30]. Hartigan and Johnson [31] have found that GSK-3*β* activity can be improved as the increase of intracellular calcium ion concentration, thus sequentially increasing the level of p-Tau. In addition, miR-12-3p with the function of direct targeting to CAV-1 can suppress abnormal hyperphosphorylation of Tau by regulating CAV-1-PI3K/Akt/GSK3*β* signal pathway in AD [32]. However, whether RSBFL-induced upregulation of CAV-1 can affect GSK-3*β* activity is not clear. Through our systematic investigations, in AD hippocampal neurons, A*β*1–42 could result in the fracture of a large of number of protrusions, serious disintegration of cell bodies, and poor growth state. On the other hand, RSBFL treatment can execute the protective role in hippocampal neurons subjected to A*β*1–42 induction, thus greatly improving cell growth state, delaying cell senescence and apoptosis through inhibiting GSK-3*β* activity. At the same time, RSBFL treatment can reduce abnormal phosphorylation of Tau protein at Thr231 and Ser9 sites, which further suggests the function of RSBFL to suppress excessive abnormal phosphorylation of Tau and reduce the expression level of p-Tau in AD cells. All of these results indicate that RSBFL may accomplish the protection of hippocampal neurons and exert normal physiological functions through modulating the balance between phosphorylation and dephosphorylation, maintaining the stability of microtubules, and regulating the balance of cytoskeleton. New evidence suggests that endoplasmic reticulum (ER) stress will cause extensive phosphorylation of Tau protein. The excessive abnormal phosphorylation of Tau protein will also cause ER stress, which is a pathological feedback loop [33]. Therefore, the antioxidant activity of Chinese herbs may have a therapeutic role in AD. Therefore, RSBFL may upregulate CAV-1 expression and inhibit GSK-3*β* activity, thereby reducing excessive abnormal phosphorylation of Tau protein and executing the neuroprotective role in AD.

In summary, upregulating CAV-1 expression, reducing GSK-3*β* activity, and inhibiting excessive abnormal phosphorylation of Tau protein may be one of the molecular mechanisms of RSBFL for the prevention and treatment of AD. In addition, whether RSBFL can execute the prevention and treatment of AD through suppressing neuronal apoptosis in hippocampal tissues needs to be further explored.
